# Comparable Effects of Sleeve Gastrectomy and Roux-en-Y Gastric Bypass on Basal Fuel Metabolism and Insulin Sensitivity in Individuals with Obesity and Type 2 Diabetes

**DOI:** 10.1155/2022/5476454

**Published:** 2022-12-21

**Authors:** Katrine Brodersen, Michael F. Nielsen, Bjørn Richelsen, Esben S. Lauritzen, Einar Pahle, Jan Abrahamsen, Bolette Hartmann, Jens J. Holst, Niels Møller

**Affiliations:** ^1^Department of Surgery, Viborg Regional Hospital, Denmark; ^2^Department of Endocrinology and Internal Medicine, Aarhus University Hospital, Denmark; ^3^Steno Diabetes Center Aarhus, Aarhus University Hospital, Denmark; ^4^Medical/Steno Aarhus Research Laboratory, Department of Clinical Medicine, Aarhus University, Denmark; ^5^Department of Surgery, University Hospital of Southern Denmark, Denmark; ^6^Department of Clinical Physiology, Viborg Regional Hospital, Denmark; ^7^Department of Biomedical Sciences and Novo Nordisk Foundation Center for Basic Metabolic Research, University of Copenhagen, Denmark

## Abstract

**Aim:**

Bariatric surgery improves insulin sensitivity and glucose tolerance in obese individuals with type 2 diabetes (T2D), but there is a lack of data comparing the underlying metabolic mechanisms after the 2 most common surgical procedures Roux-en-Y gastric bypass surgery (RYGB) and sleeve gastrectomy (SG). This study was designed to assess and compare the effects of RYGB and SG on fuel metabolism in the basal state and insulin sensitivity during a two-step euglycemic glucose clamp.

**Materials and Methods:**

16 obese individuals with T2D undergoing either RYGB (*n* = 9) or SG (*n* = 7) were investigated before and 2 months after surgery, and 8 healthy individuals without obesity and T2D served as controls. All underwent a 2 h basal study followed by a 5 h 2-step hyperinsulinemic euglycemic glucose clamp at insulin infusion rates of 0.5 and 1.0 mU/kg LBM/min.

**Results:**

RYGB and SG induced comparable 15% weight losses, normalized HbA1c, fasting glucose, fasting insulin, and decreased energy expenditure. In parallel, we recorded similar increments (about 100%) in overall insulin sensitivity (*M*-value) and glucose disposal and similar decrements (about 50%) in endogenous glucose production and FFA levels during the clamp; likewise, basal glucose and insulin concentrations decreased proportionally.

**Conclusion:**

Our data suggest that RYGB and SG improve basal fuel metabolism and two-step insulin sensitivity in the liver, muscle, and fat and seem equally favourable when investigated 2 months after surgery. This trial is registered with NCT02713555.

## 1. Introduction

Bariatric surgery is the most efficient treatment of obesity and type 2 diabetes (T2D) and produces long-term weight loss, diabetes remission, and reduced mortality [1–3].

The most frequently applied bariatric procedures worldwide are Roux-en-Y gastric bypass (RYGB) and sleeve gastrectomy (SG) [4, 5]. Recent studies indicate that RYGB is associated with 5-8% higher weight loss compared to SG [6, 7]. T2D remission rates of 48-84% (depending on definitions) have been reported following surgery, either comparably between the two types of surgery or slightly higher following RYGB [6–10]. The mechanisms behind the rapid improvement in insulin-glucose homeostasis after these surgical procedures have been debated but are still not fully understood.

The pathogenesis of T2D in obesity is linked to insulin resistance in muscle, liver, and adipose tissues, leading to decreased glucose disposal and excessive endogenous glucose production (EGP) and lipolysis; when combined with a relative *β*-cell failure with deficient insulin secretion, T2D may ensue [11, 12].

The improvement in glucose tolerance following RYGB and SG is associated with improved insulin action, which presents almost immediately after surgery before any major weight loss but shows further improvement later and in parallel with the magnitude of the weight loss [13–16]. Insulin sensitivity has been compared between the two surgical procedures employing the Matsuda index or the *M*-value from a hyperinsulinemic euglycemic clamp in insulin-resistant individuals to assess peripheral insulin sensitivity and with the homeostatic model assessment (HOMA) in individuals with T2D to assess hepatic insulin sensitivity, yielding conflicting results [8, 10, 16, 17]. It has been suggested that improved hepatic insulin sensitivity may be responsible particularly for the early recovery of glucose intolerance weeks after surgery [14, 18]. However, there is a lack of studies comparing insulin action after the two interventions in individuals with obesity-induced T2D by combining gold standard 2-step hyperinsulinemic glucose clamps with isotope dilution methodology and including a healthy control group. This approach allows definition of both peripheral and hepatic insulin sensitivity at two distinct insulin levels.

The current study was designed to assess and compare the effects of RYGB and SG on fuel metabolism in the basal state as well as hepatic and peripheral insulin sensitivity determined during a two-step euglycemic glucose clamp.

## 2. Materials and Methods

### 2.1. Participants and Study Design

#### 2.1.1. Participants

Twenty-two individuals preparing for bariatric surgery were consecutively recruited from the departments of endocrinology at Aarhus University Hospital and Viborg Regional Hospital. Two individuals were excluded due to high fasting p-glucose, 3 participants chose not to have surgery, and 1 participant left the study following surgery for personal reasons. Thus, 16 individuals completed the study days as described below.

The indication for bariatric surgery was BMI above 35 kg/m^2^, combined with T2D. T2D was defined as HbA1c ≥ 48 mmol/mol or treatment with antidiabetic agents for T2D. At recruitment, participants had started the preoperative weight loss program of 8% of the body weight in order to qualify for surgery (recommended in Denmark). The type of surgery was decided in collaboration between the patient and the endocrinologist. Eight healthy individuals were recruited by local advertisement.

Diabetes remission at 2-month follow-up was defined as HbA1c < 48 mmol/mol without antidiabetic medication or HbA1c < 42 mmol/mol with metformin therapy [19].

Prior to inclusion, obese individuals and healthy individuals were screened with a medical interview, physical examination, blood samples, and electrocardiography. Predefined criteria excluded type 1 diabetes mellitus and other significant endocrine, cardiac, or kidney disease. All individuals gave informed consent prior to inclusion in the study.

#### 2.1.2. Study Design

**Figure 1:**
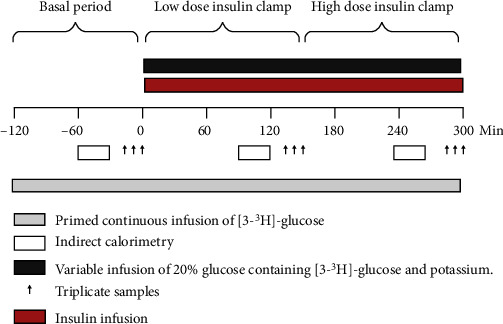
Graphical illustration of the two-step hyperinsulinemic euglycemic clamp. Abbreviations: LBM: lean body mass.

All individuals were studied before and 2 months after surgery with a dual-energy X-ray absorptiometry (DXA) and with a 2 h basal period followed by a stepwise hyperinsulinemic euglycemic clamp (HEC) with indirect calorimetry (Figure 1). Healthy individuals were studied once. HEC studies were conducted at Aarhus University Hospital, Denmark. Surgery was performed at the Surgical Department at Viborg Regional Hospital by an experienced team. Three days before each study day, individuals were instructed to continue an isocaloric diet and avoid strenuous physical activity and alcohol consumption. Metformin was paused 7 days prior to the study days, and all other medication was paused for a period of 5 times their half-life before the investigation.

The Central Denmark Region Committees on Biomedical Research Ethics approved the study (ID: 1-10-72-363-15). The study was registered at ClinicalTrials.gov (NCT02713555) and at the Danish Data Protecting Agency and was conducted in accordance with the Declaration of Helsinki.

### 2.2. Surgical Procedures

Procedures were standardized laparoscopic surgery. RYGB entailed creating a ~30 ml gastric pouch, a 60 cm long biliary limb, and a 150 cm long alimentary limb. During SG, the partial gastrectomy was performed aided by a 35 French bougie.

### 2.3. Hyperinsulinemic Euglycemic Clamp (Figure 1)

After an overnight fasting, two intravenous catheters were inserted, one in an antecubital vein and one in a dorsal heated hand vein. A primed continuous infusion of [3-^3^H]-glucose was initiated, and after 2 hours, blood samples were drawn in triplicate with 10 min intervals apart each sample. This period was defined as the basal period.

The insulin clamp was initiated with an insulin infusion of 0.5 mU/kg LBM/min (time = 0 min). In an attempt to maintain glucose-specific activities stable, the infusion rate of [3-^3^H]-glucose was reduced by 50% [20]. Steady-state samples during low-dose insulin infusion were drawn with 10 min interval (time = 130 − 150). The insulin infusion was increased to 1.0 mU/kg LBM/min while the infusion of [3-^3^H]-glucose was reduced by another 50% [20]. Steady-state samples during the high-dose insulin infusion were obtained at 10 min intervals from 270 to 300 min.

Throughout the clamp steps, plasma glucose was held at ≈5 mmol/l with a variable infusion of 20% glucose also containing [3-^3^H]-glucose and potassium (hot GINF). Calculations of EGP, rate of glucose appearance (Ra), rate of glucose disappearance (Rd), and the *M*-value were performed as previously described [21].

### 2.4. Body Composition

Changes in anthropometric parameters were determined using a DXA scanner (Horizon, Hologic, MA, USA).

### 2.5. Indirect Calorimetry

Energy expenditure (EE), nonoxidative glucose disposal, and oxidation rates of lipids (LIOX) and glucose (CHOX) were calculated based on ~20-minute collection of respiratory gasses with Deltatrac II calorimeter (Helsinki, Finland). Measurements were obtained prior to steady state during the HEC at *t* = −60 minutes, 90 minutes, and 230 minutes as previously described [22].

### 2.6. Analytical Methods

Plasma glucose concentrations were measured on the study day using YSI 2300 model Stat Plus (Bie & Berntsen, Denmark). Serum insulin and C-peptide concentrations were measured with enzyme-linked immunosorbent assay (ELISA) kits (cat no. 10-1113-01 and 10-1136-01, Mercodia, Sweden). Glucagon was measured using a C-terminally directed antiserum (code no. 4305) measuring glucagon of pancreatic origin as previously described [23]. Sensitivity for the assay was below 1 pmol/l and intra-assay coefficient of variation below 10%. Serum FFA concentrations were analyzed using in vitro enzymatic colorimetric method assay NEFA-HR(2) (Nefa-HR R1 set 434-91795 and R2 set 436-91995, Wako Chemicals GmbH, Germany). Assays were carried out in accordance with the manufacturer's instructions, and all quality controls were within required limits.

### 2.7. Statistical Analysis

Data were analyzed using a multilevel linear mixed model with group (RYGB and SG), visit (pre- and postoperative), and insulin levels (basal, low insulin, and high insulin) and the interaction between them as fixed effects and with study participants as a random effect. The assumptions underlying the multilevel mixed model were validated by inspection of quantile-quantile plots of the standardized residuals and by inspection of scatter plots of the standardized residuals and the fitted values. If assumptions were not fulfilled, the data were log transformed.

Normally distributed data are presented as means ± standard error of the mean (SEM), while not normally distributed data are presented as medians (95% confidence interval), unless otherwise specified. A *p* value < 0.05 was considered significant.

Three-way and two-way interactions are available in the Supplementary Appendix A as Table A1: interactions.

#### 2.7.1. Power Calculations

Based on previous studies with an intrapersonal *M*-value SD of 5% after repeated clamps and a detection level of 8%, it can be calculated that a minimum of 7 participants should be included in both groups.

## 3. Results

### 3.1. Participants (Table 1)

Nine individuals underwent RYGB, 7 individuals underwent SG, and 8 healthy individuals were studied as controls (CTS).

As shown, the gender ratio was not equally distributed which is reflected in the tendency to higher body weight and lean body mass and lesser fat mass in the SG group as compared with the RYGB group. In the bariatric groups, all individuals were obese and had T2D with similar HbA1c, 51.8 mmol/mol (RYGB) versus 52.9 mmol/mol (SG). All individuals received metformin prior to surgery. In the RYGB group, 2 individuals additionally received a DPP-4 inhibitor, 1 individual a GLP-1 agonist, and 1 individual an SGLT2 inhibitor. In the SG group, 2 individuals received SGLT2 inhibitors. Prior to inclusion, individuals who later underwent RYGB had lost 4.8 kg ± 1.7 (*p* < 0.05), and individuals who later underwent SG had lost 6.2 kg ± 2.0 (*p* < 0.05) from first contact to the outpatients' clinic to inclusion. There was no difference between groups.

### 3.2. Changes in Anthropometric Parameters

After 2 months, surgery decreased body weight by 15% (range, 13%-18%) (*p* < 0.01) following RYGB and by 15% (range, 12%-18%) (*p* < 0.01) after SG, and BMI decreased by 6.2 kg/m^2^ ± 0.7 (*p* < 0.01) without any difference between the regimens. Fat mass decreased by 10.3 kg ± 1.2 (*p* < 0.01) following RYGB and by 12.6 kg ± 1.3 (*p* < 0.01) after SG, resulting in a comparable fat mass following surgery. LBM decreased by 5.5 kg ± 1.1 (*p* < 0.01) following RYGB and by 7.1 kg ± 1.3 (*p* < 0.01) after SG, and groups remained comparable. SG decreased triglyceride levels by 44% (*p* < 0.05) to 2.1 mmol/l (range, 0.9-3.8), and RYGB decreased levels by 35% to 1.6 mmol/l (range, 1.1-2.4). After surgery, the two groups were comparable.

HbA1c decreased similarly by 26% to 39 mmol/mol (range, 38.1-44.0) (*p* < 0.01) after SG and 21% to 41 mmol/mol (range, 38.1-44.0) (*p* < 0.01) after RYGB, and all participants discontinued their antidiabetic medication except for 1 individual in the RYGB group and 2 individuals in the SG group who remained on metformin. All participants in the two surgical groups were in diabetes remission at 2 months according to the aforementioned definition.

### 3.3. Basal Fuel Metabolism

#### 3.3.1. Glucose Turnover

**Figure 2:**
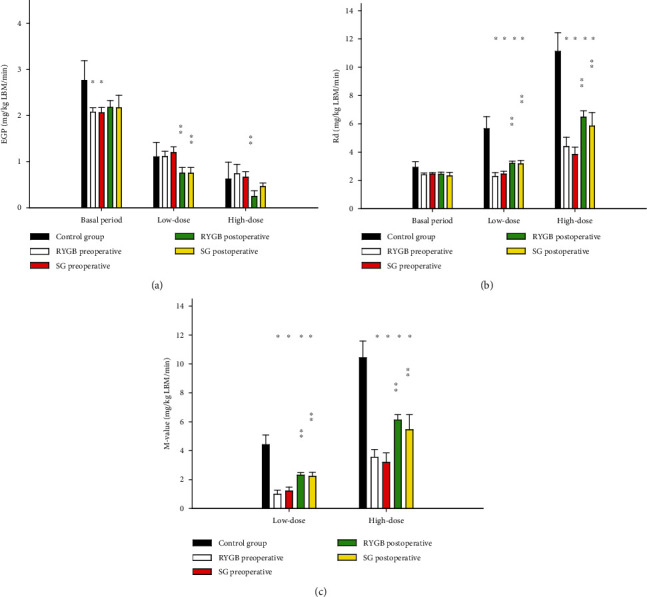
EGP, RD, and *M*-values. Displayed as the mean with the standard error of the mean. *p* values < 0.05 are considered statistical significant. ^∗^Significant difference from the control group. ^∗∗^Significant difference between post- and preoperative values within each group. ^∗∗∗^Significant difference between RYGB and SG preoperative. Abbreviations: Rd: rate of disappearance; EGP: endogen glucose production; low dose: low-dose insulin infusion; high dose: high-dose insulin infusion.

During the basal period, EGP and Rd were unaffected by surgery and the type of surgery (Figures 2(a) and 2(c)). EGP was higher in healthy CTS compared to obese individuals with T2D prior to surgery (RYGB vs. CTS, *p* < 0.05; SG vs. CTS, *p* < 0.05, Figure 2(a)) while Rd was comparable between CTS and surgical groups (*p* = 0.38, Figure 2(c)).

#### 3.3.2. Hormone and Substrate Concentrations

**Figure 3:**
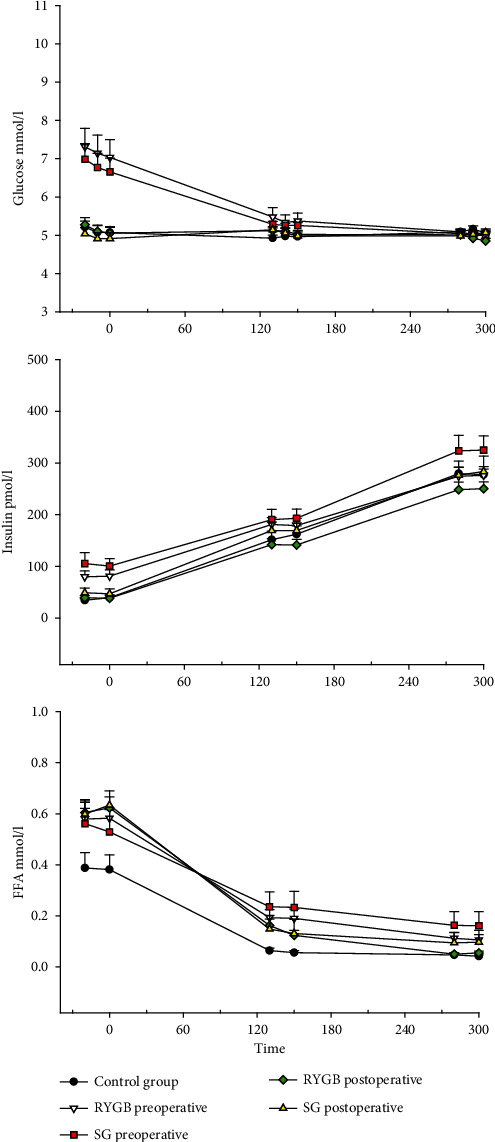
Glucose, insulin, and free fatty acids during the basal period and two-step hyperinsulinemic euglycemic clamp. Displayed as the mean with the standard error of the mean. *p* values < 0.05 are considered statistical significant. ^∗^Significant difference from the control group. ^∗∗^Significant difference between post- and preoperative values within each group. ^∗∗∗^Significant difference between RYGB and SG. Abbreviations: FFA: free fatty acids.

After surgery, insulin levels decreased by about 50 pmol/l (*p* < 0.01) and glucose levels by 28% (*p* < 0.01) regardless of type of surgery, and C-peptide decreased by 436 pmol/l ± 64 (*p* < 0.01), remaining comparable between groups (Figure 3). Insulin and C-peptide levels were higher before surgery compared to CTS and improvements after surgery without group differences. Following SG, glucagon levels decreased by 2.1 pmol/l ± 0.6 (*p* < 0.011) and tended to decrease following RYGB (*p* = 0.06). In addition, FFA levels were not affected by either type of surgery but FFA levels remained about 25% higher compared to the CTS (RYGB, *p* < 0.05; SG, *p* < 0.05) (Figure 3).

#### 3.3.3. Substrate Utilization (Table 2)

During the basal period, nonoxidative glucose disposal rates were unaffected by either type of surgery (*p* = 0.25) but tended to be higher compared to CTS (RYGB, *p* = 0.11; SG, *p* = 0.06).

Carbohydrate oxidation (CHOX) tended to decrease following SG (*p* = 0.07), and levels remained 4 times higher in CTS compared to SG (*p* < 0.01) and 2 times higher compared to RYGB (*p* < 0.01) during basal states.

Lipid oxidation (LIOX) tended to increased slightly following both types of surgery (SG, *p* = 0.09; RYGB, *p* = 0.06). However, levels remained 2-3 times lower in CTS compared to RYGB (*p* < 0.01) and SG (*p* < 0.01).

### 3.4. Glucose Turnover and Insulin Sensitivity during Hyperinsulinemic Clamp

#### 3.4.1. Glucose Turnover during HEC

Following both types of surgery, hepatic insulin sensitivity increased as EGP decreased by 0.4 mg/kg LBM/min during low insulin infusion (RYGB: *p* < 0.01; SG: *p* > 0.05). During high-dose insulin infusion, EGP decreased by 0.4 mg/kg LBM/min following RYGB (*p* < 0.05) and by 0.3 mg/kg LBM/min following SG (*p* = 0.06). Levels remained comparable between groups (Figure 2(a)).

Peripheral insulin sensitivity also improved with an increase in Rd by 33% following SG (*p* < 0.05) and 47% after RYGB during low-dose insulin infusion (*p* < 0.05) and 45% following SG (*p* < 0.01) and 61% after RYGB (*p* < 0.01) during high-dose insulin infusion, without any difference between type of surgery. However, Rd remained 39% lower following RYGB and 50% lower following SG (*p* < 0.01) compared to CTS (Figure 2(c)).

Overall insulin sensitivity increased in terms of *M*-values by 1.18 mg/kg LBM/min ± 0.35 during low-dose insulin infusion (*p* < 0.05) and by 2.44 mg/kg LBM/min ± 0.35 during high-dose insulin infusion (*p* < 0.01), without any difference between types of surgery. *M*-values increased towards the levels of CTS but remained lower (Figure 2(b)).

#### 3.4.2. Hormone and Substrate Concentrations during HEC

Insulin levels increased by 110 pmol/l from low insulin infusion to high insulin infusion, in all groups (Figure 3). However, after surgery, insulin levels were 33 pmol/l ± 15.6 lower following SG (*p* < 0.05) and decreased insignificantly by 20 pmol/l ± 14.4 following RYGB (*p* = 0.17). Nonetheless, glucose and insulin levels were comparable between RYGB, SG, and CTS during low- and high-dose insulin infusions both before and after surgery. C-peptide levels decreased from each insulin step (*p* < 0.05). Surgery did not decrease C-peptide levels.

Glucagon levels decreased after SG during both insulin steps (*p* < 0.05) and tended to do so during low-dose insulin infusion after RYGB (*p* = 0.08). Levels were comparable after surgery as well as compared to CTS. Insulin suppressed FFA levels to a comparable extent in RYGB and SG groups during both low- and high-dose insulin infusions. The levels of FFA decreased after RYGB (27%, *p* = 0.09) and decreased as well following SG (3%, *p* < 0.05) during low-dose insulin infusion. Moreover, FFA levels decreased by 42% after RYGB (*p* < 0.01) and 55% following SG (*p* < 0.01) during high-dose insulin infusion. However, FFA suppression remained impaired compared to CTS with 220% higher levels following RYGB (*p* < 0.05) and 185% higher levels following SG (*p* = 0.06) during low-dose insulin infusion. While FFA levels were 2-3 times higher prior to surgery (*p* < 0.02), they were comparable to CTS following both types of surgery during high-dose insulin infusion (Figure 3).

#### 3.4.3. Substrate Utilization during HEC (Table 2)

Nonoxidative glucose disposal increased following both types of surgery during low- and high-dose insulin infusions (*p* < 0.01), and these rates were comparable following RYGB and SG. But while rates were comparable between CTS and SG during low-dose insulin infusion, there was a tendency to lower levels following RYGB compared to CTS (*p* = 0.07), and levels were 50% lower following both types of surgery during high-dose insulin infusion compared to CTS (*p* < 0.01).

CHOX was unaffected by surgery during insulin infusions in either group. While CHOX was comparable between basal states and low-dose insulin infusion in the surgical groups preoperatively, CHOX increased after insulin infusion as expected postoperatively (*p* < 0.01). However, levels of oxidation remained more than 35% higher in CTS compared to RYGB (*p* < 0.01) and 60% compared to SG during low and high insulin infusion (*p* < 0.01). CHOX was 35% mg/kg LBM/min higher in high-dose insulin infusion following RYGB compared to SG (*p* < 0.05).

LIOX increased slightly following surgery but only in low-dose insulin infusion states (*p* = 0.01). Furthermore, LIOX was comparable between low-dose and high-dose insulin infusions prior to surgery (*p* = 0.18) while LIOX decreased with increasing insulin infusion after surgery (*p* < 0.01). However, levels remained 70% lower during insulin infusion in the CTS group compared to RYGB (*p* < 0.05) and 75% lower compared to SG (*p* < 0.01).

## 4. Discussion

The main findings of the present study include normalization of hepatic and adipose tissue insulin sensitivity and a close to 100% improvement of peripheral (muscle) insulin sensitivity following both SG and RYGB. These observations were recorded at 2 different insulin levels and were largely indistinguishable between the 2 treatment groups. A very recent publication reported a comparable close to 100% increase in peripheral insulin sensitivity (*M*-value) in less obese subjects (BMI 27.5-32.5 kg/m^2^) with T2D, investigated 6 months after both SG and RYGB [24]. Since the seminal observations by Randle et al. in 1963 [25], it has been broadly recognized that increased lipolysis and high levels of FFA and other lipid intermediates via lipotoxic effectors play a key role in inducing insulin resistance, although the precise intracellular mechanisms remain elusive [26–29]. It is thus highly plausible that lipotoxicity and perhaps low-grade inflammation have been instrumental in generating aberrant tissue-specific insulin resistance in muscle, liver, and adipose tissues preoperatively and that these factors have been curtailed by the postoperative loss of ectopic fat in the obese individuals.

Caloric restriction—*per se*—causes a decrease in intrahepatic triglyceride content which is associated with decreased EGP via yet unknown mechanisms [30]. In parallel, the loss of intrahepatic triglyceride restores insulin clearance [31]. Thus, the improvement in hepatic insulin sensitivity and the increased insulin clearance in our study are most likely due to the reduction in appetite, decreased food intake, and weight loss, inflicted by both types of surgery. However, RYGB has been shown to reduce intrahepatic lipid content to a greater extent than very-low-calorie diet, and decreasing intrahepatic lipid accumulation is correlated with GLP-1 peak levels [32, 33]. GLP-1 receptor agonists' treatment reduces intrahepatic lipid content, induces a small weight loss, and improves glucose tolerance in individuals with T2D [34, 35], following RYGB and SG GLP-1 levels that increased similarly, thereby enhancing the beneficial effects of caloric restriction on the liver [10].

In contrast to previous studies in individuals with T2D, we found a lower EGP in individuals with obesity and T2D compared to healthy individuals. This apparently contradictory finding most likely relates to the weight loss prior to baseline study days, and hepatic insulin sensitivity may well already have improved in this process, as previously observed [14]. Very few studies have compared EGP during ambient glycemic conditions to a healthy control group; one study reported 30–40% increased endogenous glucose production in preoperative diabetic subjects at an ambient glucose level of 7.6 mM [36].

Both types of surgery decreased energy expenditure (supplementary appendix B) during the basal period increased lipid oxidation, which is expected in relation to weight loss and may reflect loss of thermoactive muscle and fat mass [37] and decreased insulin levels. Glucose utilization and nonoxidative glucose disposal increased with insulin infusion, and FFA levels decreased after surgery indicating increased insulin sensitivity. However, still compared to healthy individuals, glucose oxidation was lower and lipid oxidation higher following surgery. Although these changes were recorded at 2 months, it should be mentioned that evidence of initial improvement in hepatic insulin sensitivity and later improvements in peripheral insulin sensitivity in parallel with the weight loss induces improvements in glucose tolerance. And these changes are more or less stationary at 12 and 24 months after RYGB [14, 38] implicating that the alterations are more or less permanent.

This study has obvious limitations. First, its sample size was relatively small, and the participants were not randomly assigned to the bariatric procedures which may induce a risk of statistical type 2 error and selection bias. Regarding the risk of a type 2 error, the overall statistically significant findings from the HEC in our opinion indicate that we have sufficient power to detect clinically relevant changes following surgery; yet more subtle differences between the two surgical procedures may have been missed. Regarding selection bias, the two groups were largely comparable (Table 1) except as regards to gender (see below). Second, it should be noted that higher T2D relapse rates have been observed in females compared to males in a retrospective study of bariatric surgery [39]. Even so, a prospective pair-matched cohort study found no differences in weight loss or T2D remission between sexes [40]. In addition, comparable insulin resistance in the muscle and liver has been observed following lipid challenges in men and women indicating that the underlying pathology in relation to insulin sensitivity and glucose turnover is comparable between genders [29]. Third, the individuals with T2D and obesity were not weight stable prior to surgery. This may have implications for the comparisons with the healthy control group, but since these initial weight losses were similar between the two groups, the comparisons between the two groups are valid. Finally, insulin levels during HEC were lower following surgery due to an increased insulin clearance [41], implying that the observed improvements of insulin sensitivity may be underestimated.

In conclusion, SG resulted in a normalized hepatic insulin sensitivity, increased insulin clearance, and an improved peripheral insulin sensitivity comparable to RYGB. Concomitantly, with the large weight loss, lipid oxidation increased and glucose utilization shifted towards increased nonoxidative glucose disposal. Thus, SG seems to offer the same beneficial metabolic effects as RYGB at 2 months following surgery.

## Figures and Tables

**Table 1:** Baseline characteristics.

	CTS	RYGB	SG	*p* value
	*n* = 8	*n* = 9	*n* = 7	
Female sex, no. (%)	4 (50)	9 (100)	3 (43)	*p* < 0.05^∗^^,^^∗∗∗^
Age (years)	49.3 (27 66)	59.7 (37 58)	47.4 (39 51)	
T2D duration (years)	—	2.8 (0.4 6)	1.5 (0.1 4)	
Weight (kg)	74.1 (59 98)	117.3 (94 144)	126.8 (95 149)	*p* < 0.01^∗^^,^^∗∗^
BMI (kg/m^2^)	25.1 (23 27)	41.9 (33 50)	41.6 (35 48)	*p* < 0.01^∗^^,^^∗∗^
LBM (kg)	49.9 (35 68)	60.5 (52 72)	71.7 (50 88)	*p* < 0.05^∗^^,^^∗∗^
FM (kg)	24.2 (21 33)	54.9 (36 77)	53 (44 64)	*p* < 0.01^∗^^,^^∗∗^
FM (%)	32.5 (26 38)	46.5 (38 54)	42 (35 51)	*p* < 0.01^∗^^,^^∗∗^
HbA1c (mmol/mol)	35.25 (28 40)	51.8 (45 59)	52.9 (44 64)	*p* < 0.01^∗^^,^^∗∗^

Data are expressed as mean (range). For comparisons, an analysis of variance (ANOVA) or a nonparametric Kruskal-Wallis test was applied. Post hoc tests were performed when appropriate, with an unpaired *T*-test. For dichotomous values, we used Fischer's exact test. Comparisons: ^∗^CTS vs. RYGB, ^∗∗^CTS vs. SG, and ^∗∗∗^RYGB vs. SG. Abbreviations: CTS: control subjects; RYGB: Roux-en-Y gastric bypass group; SG: sleeve gastrectomy group; BMI: body mass index; LBM: lean body mass; FM: fat mass; HbA1c: glycated haemoglobin.

**Table 2:** Indirect calorimetry.

		CTS	RYGB pre	RYGB post	SG pre	SG post
Basal		*n* = 8	*n* = 9	*n* = 9	*n* = 6	*n* = 7
CHOX	mg/kg LBM/min	2.92 ± 0.40	1.45 ± 0.11	1.12 ± 0.09	1.38 ± 0.17	0.69 ± 0.30
LIOX	mg/kg LBM/min	0.48 ± 0.18	1.03 ± 0.09	1.12 ± 0.09	1.07 ± 0.16	1.37 ± 0.10
Non-ox glucose storage	mg/kg LBM/min	0.01 ± 0.54	0.95 ± 0.13	1.22 ± 0.31	1.05 ± 0.21	1.63 ± 0.28

Low insulin		*n* = 7	*n* = 9	*n* = 9	*n* = 6	*n* = 7
CHOX	mg/kg LBM/min	3.56 ± 0.27	1.82 ± 0.33	1.73 ± 0.15	1.49 ± 0.27	1.27 ± 0.25
LIOX	mg/kg LBM/min	0.28 ± 0.13	0.60 ± 0.13	0.93 ± 0.06	0.96 ± 0.21	1.10 ± 0.10
Non-ox glucose storage	mg/kg LBM/min	2.09 ± 0.78	0.46 ± 0.32	1.48 ± 0.22	0.94 ± 0.30	1.90 ± 0.33

High insulin		*n* = 8	*n* = 9	*n* = 9	*n* = 6	*n* = 7
CHOX	mg/kg LBM/min	4.59 ± 0.33	2.45 ± 0.21	2.90 ± 0.16	2.00 ± 0.20	2.21 ± 0.20
LIOX	mg/kg LBM/min	0.04 ± 0.12	0.57 ± 0.09	0.46 ± 0.07	0.72 ± 0.13	0.71 ± 0.15
Non-ox glucose storage	mg/kg LBM/min	6.84 ± 1.18	1.95 ± 0.49	3.58 ± 0.44	1.66 ± 0.49	3.64 ± 0.96

Carbohydrate and lipid oxidation rates and nonoxidative glucose storage and during basal conditions, low insulin infusion (0.5 mU/kg LBM/min), and high insulin infusion (1 mU/kg LBM/min) during a hyperinsulinemic euglycemic clamp. Data are expressed as mean ± SEM. Abbreviations: CTS: control subjects; RYGB: Roux-en-Y gastric bypass group; SG: sleeve gastrectomy group; pre: preoperatively; post: postoperatively; CHOX: carbohydrate oxidation; LIOX: lipid oxidation; Non-ox glucose storage: nonoxidative glucose storage; LBM: lean body mass.

## Data Availability

The data that support the findings of this study are available from the corresponding author upon reasonable request.

## Supplementary Materials

Supplementary 1 Table A1: interactions from the multilevel mixed effects linear regression model on Roux-en-Y gastric bypass and sleeve gastrectomy group.

Supplementary 2 Supplementary Appendix B: energy expenditure during basal conditions, low insulin infusion (0.5 mU/kg LBM/min), and high insulin infusion (1 mU/kg LBM/min) during a hyperinsulinemic euglycemic clamp.
