# Supplementary material for: Comparable Effects of Sleeve Gastrectomy and Roux-en-Y Gastric Bypass on Basal Fuel Metabolism and Insulin Sensitivity in Individuals with Obesity and Type 2 Diabetes
Source: J Diabetes Res. 2022 Dec 21;2022:5476454. doi: 10.1155/2022/5476454 (PMC9798105; doi:10.1155/2022/5476454)
Supplement: Supplementary 1 — Table A1: interactions from the multilevel mixed effects linear regression model on Roux-en-Y gastric bypass and sleeve gastrectomy group. [file 5476454.f1.pdf]

	Analysis comparing RYGB and SG				Analysis comparing Control group, RYGB and SG			
	Three-way interaction	Two-way interaction			Three-way interaction	Two-way interaction		
	Group##Visit##Time	Time#Group	Visit#Group	Time#Visit	Group##Visit##Time	Time#Group	Visit#Group	Time#Visit
Endogen glucose production	0.4884	0.8883	0.7111	0.0038	0.6539	0.0205	0.7258	0.0312
Rate of appereance	0.756	0.3033	0.3726	0.0069	0.7612	0.0001	0.3804	0.0073
Rate of disappereance	0.9579	0.3523	0.3406	0.0015	0.958	0.0000	0.3603	0.0013
M-value	0.9702	0.2600	0.5543	0.0170	0.9717	0.0000	0.5752	0.0219
Glucose	0.9489	0.495	0.6681	0.000	0.9443	0.0000	0.6507	0.0000
Insulin (Low-Dose, High-Dose)	0.2791	0.642	0.4618	0.9828	0.2667	0.8718	0.4519	0.9824
c-peptide	0.9322	0.8899	0.4573	0.0000	0.9249	0.0000	0.4433	0.0000
FFA (Low-Dose, High-Dose)	0.6544	0.3005	0.8783	0.0101	0.2217	0.0072	0.8156	0.7243
Glucagon	0.965	0.1173	0.1025	0.3573	0.959	0.2362	0.089	0.3069
Non-oxidativ glucose storage	0.8901	0.4799	0.6249	0.0148	0.9181	0.0000	0.6091	0.042
Charbohydrate oxidation	0.7958	0.563	0.4256	0.0115	0.8403	0.1808	0.4714	0.0307
Lipid oxidation	0.2232	0.6809	0.6661	0.0264	0.2457	0.8528	0.6742	0.0319
Energy expenditure	0.4087	0.5351	0.5632	0.2537	0.4383	0.1269	0.5697	0.285

Table A1. Interactions from the multilevel mixed effects linear regression model on Roux-en-Y Gastric Bypass and Sleeve Gastrectomy group. Main analysis marked blue: Factors: 1. Group (RYGB and SG), 2. Visit (pre and postoperative) 3. Time (insulin level during hyperinsulineamic euglycemic clamp). Secondary comparison to control group marked green: Factors: 1. Group (Control, RYGB and SG), 2. Visit (pre and postoperative) 3. Time (insulin level during hyperinsulineamic euglycemic glucose clamp).
