# Supplementary material for: Comparable Effects of Sleeve Gastrectomy and Roux-en-Y Gastric Bypass on Basal Fuel Metabolism and Insulin Sensitivity in Individuals with Obesity and Type 2 Diabetes
Source: J Diabetes Res. 2022 Dec 21;2022:5476454. doi: 10.1155/2022/5476454 (PMC9798105; doi:10.1155/2022/5476454)
Supplement: Supplementary 2 — Supplementary Appendix B: energy expenditure during basal conditions, low insulin infusion (0.5 mU/kg LBM/min), and high insulin infusion (1 mU/kg LBM/min) during a hyperinsulinemic euglycemic clamp. [file 5476454.f2.pdf]

Supplementary Appendix B:

Energy expenditure

EE decreased ~ 230 kcal/24 hour ($p < 0.01$) following surgery in both groups ($p < 0.01$). Levels were comparable between RYGB and CTS but tended to be higher following SG compared to CTS ($p > 0.08$).

Table B1

Energy expenditure during indirect calorimetry

		CTS	RYGB pre	RYGB post	SG pre	SG post
**Basal**		**n = 8**	**n = 9**	**n = 9**	**n = 6**	**n = 7**
Energy Expenditure	kcal/24h	1463 ± 111	1692 ± 79	1444 ± 60	1980 ± 132	1699 ± 101
**Low insulin**		**n = 7**	**n = 9**	**n = 9**	**n = 6**	**n = 7**
Energy Expenditure	kcal/24h	1421 ± 127	1595 ± 64	1443 ± 47	1992 ± 157	1684 ± 98
**High insulin**		**n = 8**	**n = 9**	**n = 9**	**n = 6**	**n = 7**
Energy Expenditure	kcal/24h	1479 ± 116	1609 ± 75	1414 ± 38	1935 ± 151	1702 ± 101

Energy expenditure during basal conditions, low insulin infusion (0.5 mU/kg LBM/min) and high insulin infusion (1 mU/kg LBM/min) during a hyperinsulinaemic euglycemic clamp. Data are expressed as mean ± SEM. Abbreviations: CTS, control subjects; RYGB, roux-en-y gastric bypass group; SG, sleeve gastrectomy group; pre, preoperatively; post, postoperatively.
